# Optimizing Continuous Renal Replacement Therapy with Regional Citrate Anticoagulation: Insights from the ORCA Trial—A Retrospective Study on 10 Years of Practice

**DOI:** 10.3390/life14101304

**Published:** 2024-10-14

**Authors:** Rita Jacobs, Walter Verbrugghe, Jason Bouziotis, Ingrid Baar, Karolien Dams, Annick De Weerdt, Philippe G. Jorens

**Affiliations:** 1 The Departments of Critical Care Medicine, Antwerp University Hospital, 2650 Edegem, Belgiumkarolien.dams@uza.be (K.D.);; 2 Campus Drie Eiken, University of Antwerp, LEMP, Universiteitsplein 1, 2610 Antwerp, Belgium; 3 Clinical Trial Center (CTC), CRC Antwerp, Antwerp University Hospital, University of Antwerp, 2650 Edegem, Belgium

**Keywords:** citrate anticoagulation, heparin, renal replacement therapy, filter lifespan, bleeding, acid–base disturbances

## Abstract

(1) Background: Citrate is preferred in continuous renal replacement therapy (CRRT) for critically ill patients because it prolongs filter life and reduces bleeding risks compared to unfractionated heparin (UFH). However, regional citrate anticoagulation (RCA) can lead to acid–base disturbances, citrate accumulation, and overload. This study compares the safety and efficacy of citrate-based CRRT with UFH and no anticoagulation (NA) in acute kidney injury (AKI) patients. (2) Methods: A retrospective analysis was conducted on adult patients (≥18 years) who underwent CRRT from July 2010 to June 2021 in an intensive care unit. (3) Results: Among 829 AKI patients on CRRT: 552 received RCA, 232 UFH, and 45 NA. The RCA group had a longer filter lifespan compared to UFH and NA (56 h [IQR, 24–110] vs. 36.0 h [IQR, 17–63.5] vs. 22 h [IQR, 12–48]; all P_adj_ < 0.001). Bleeding complications were fewer in the RCA group than in the UFH group (median 3 units [IQR, 2–7 units] vs. median 5 units [IQR, 2–12 units]; P_adj_ < 0.001) and fewer in the NA group than in the UFH group (median 3 units [IQR, 1–5 units] vs. 5 units [IQR, 2–12 units]; P_adj_ = 0.03). Metabolic alkalosis was more common in the RCA group (32.5%) compared to the UFH (16.2%) and NA (13.5%) groups, while metabolic acidosis persisted more in the UFH group and NA group (29.1% and 34.6%) by the end of therapy vs. the citrate group (16.8%). ICU mortality was lower in the RCA group (52.7%) compared to the UFH group (63.4%; P_adj_ = 0.02) and NA group (77.8%; P_adj_ = 0.003). (4) Conclusions: Citrate anticoagulation outperforms heparin-based and no anticoagulation in filter patency, potentially leading to better outcomes through improved therapy effectiveness and reduced transfusion needs. However, careful monitoring is crucial to limit potential complications attributable to its use.

## 1. Introduction

The prevalence of acute kidney injury (AKI) in critically ill patients is high, affecting more than half of intensive care patients and leading to increased morbidity and mortality [1]. Continuous renal replacement therapy (CRRT) has significantly improved since its first implementation in 1977 and has become the modality of choice in the intensive care unit (ICU) due to its superiority compared to intermittent hemodialysis (IHD) in hemodynamically unstable patients [2,3]. To prevent clotting of the circuit during CRRT, anticoagulation is used. Regional citrate anticoagulation (RCA) is recommended as first-line anticoagulation due to its unique mechanism of action and potential benefits in maintaining filter patency and reduced risk of bleeding compared to unfractionated heparin (UFH) [4,5]. Citrate prevents clot formation by chelating calcium ions essential for the coagulation cascade [6].

Despite its increasing use, citrate remains a significant concern for many clinicians due to the potential for metabolic complications, leading to reluctance in adopting citrate anticoagulation. Citrate chelation reduces systemic calcium levels, which may lead to hypocalcemia and related complications, such as muscle cramps, arrhythmias, and hypotension. Impaired citrate metabolization, particularly in patients with liver dysfunction or severe shock, can cause citrate accumulation and acidosis. Conversely, an increased citrate load may result in metabolic alkalosis, this is the case when the body’s capacity to metabolize citrate is not reached and all citrate calcium complexes are metabolized [7]. This condition can be life-threatening, especially when pH exceeds 7.65, with mortality rates reaching up to 80%. Severe alkalosis can cause respiratory depression, impair oxygen delivery, and reduce perfusion to vital organs. Furthermore, RCA can induce electrolyte abnormalities, such as hypocalcemia, hypokalemia, and hypomagnesemia, which are critical concerns in ICU patients already vulnerable to arrhythmias, neuromuscular dysfunction, and worsening renal injury [8,9].

While numerous studies have demonstrated the advantages of RCA, and the KDIGO guidelines recommend citrate as the default anticoagulation method [3], heparin remains the most used anticoagulant in, i.e., the United States [10]. Given the limited data on the long-term use and outcomes of RCA, we conducted a retrospective review of all CRRT sessions performed in our center over more than 10 years (July 2010 until June 2021). This study aims to provide real-life data outside the scope of randomized controlled trials (RCT), investigating the efficacy and safety of RCA compared with UFH or no anticoagulation (NA) during CRRT.

## 2. Materials and Methods

### 2.1. Study Design and Setting

This retrospective single-center study compared the safety and efficacy of anticoagulant strategies for CRRT in the ICU of the Antwerp University Hospital (UZA), a 45-bed tertiary, teaching hospital in Belgium. The study (Optimizing continuous renal replacement therapy with Regional Citrate Anticoagulation, ORCA) was approved by the Health Research Ethics Committee of the Antwerp University Hospital (B.U.N.: N/A). Due to its observational and retrospective nature, the requirement for written informed consent was waived.

- Patient selection

Inclusion criteria were all adult critically ill patients (age ≥ 18 years) admitted to the ICU with AKI who underwent CRRT.

- CRRT protocol

Renal replacement therapy (RRT) was initiated in patients with AKI based on KDIGO stage 3 criteria or in cases of life-threatening changes in fluid, electrolyte, and acid–base balance. The adoption of an anticoagulation strategy, although not protocolized, was based on the clinical context and specific patient characteristics.

At the beginning of the study, heparin anticoagulation was the sole anticoagulation method, due to the unavailability of citrate anticoagulation. Subsequently, citrate anticoagulation became the preferred method according to KDIGO guidelines, especially for patients with a high risk of bleeding or those with contraindications to heparin. Heparin anticoagulation was adopted as the primary strategy for patients requiring continuous anticoagulation for other medical reasons. For patients in whom anticoagulation was potentially harmful, such as those with liver failure, anticoagulation was deemed unnecessary.

During that time frame, all CRRT sessions were performed using the Prismaflex^®^ machine (Baxter Healthcare, Deerfield, IL, USA) with an acrylonitrile 69 surface-treated (AN69ST150) membrane. Venovenous access was obtained via a double-lumen polyurethane catheter and the location of insertion was at the discretion of the attending physician. CRRT was administered according to a dedicated protocol.

For citrate anticoagulation, Prismocitrate 18/0 solution (Baxter Healthcare, Deerfield, IL, USA) was infused pre-filter at a starting dose of 3 mmol/L, with bicarbonate-buffered solutions used to achieve a dose of at least 25 mL/kg/h for all patients. Ionized calcium levels were closely monitored and adjusted according to a protocolized algorithm, targeting an ionized calcium level of 0.25–0.35 mmol/L postfilter. Calcium replacement was provided to restore systemic ionized calcium levels. Acid–base status electrolytes and ionized calcium levels were closely monitored throughout CRRT. A total calcium to ionized calcium ratio greater than 2.5 was considered indicative of citrate accumulation.

For patients receiving heparin-CRRT, UFH was administered at a starting dose of 10 IU/kg/h, aiming for an activated partial thromboplastin time (aPTT) of 45–60 s. In patients with contraindications to both citrate and heparin (e.g., bleeding, liver failure), CRRT was performed without anticoagulation (NA group). The blood flow rate was initially set at 150 mL/min and replacement solutions were delivered both pre- and post-dilution aiming at a target dose of at least 25 mL/kg/h.

### 2.2. Outcome Measures

- Primary endpoints

The primary endpoints were safety and efficacy. Efficacy was defined as the survival time in hours of the first filter. Filter clotting was identified by spontaneous clotting or a persistently high trans-membrane pressure (TMP) greater than 250 mmHg, and all data were registered in the patient data management system (PDMS). To evaluate safety, the incidence and types of complications associated with anticoagulation were recorded, including metabolic derangements, such as metabolic acidosis (pH< 7.3), metabolic alkalosis (pH > 7.5), citrate accumulation (total/ionized calcium ratio ≥ 2.5), and electrolyte disturbances such as hypocalcemia (ionized calcium < 0.85 mmol/L), hypokalemia (potassium < 3.5 mmol/L), hypomagnesemia (magnesium < 0.66 mmol/L), hypophosphatemia (phosphate < 0.78 mmol/L), hypernatremia (sodium >145 mmol/L), and hyponatremia (sodium < 130 mmol/L). Bleeding complications were also tracked: number of patients transfused, number of packed cells transfused, overt bleeding, defined as an actionable sign of hemorrhage plus a hemoglobin drop requiring an intervention by a health care professional.

- Secondary outcomes

Secondary outcomes included all-cause mortality in the ICU and during hospitalization, heparin-induced thrombocytopenia (HIT), the number of filters used during the study period regardless of the reason for cessation, and renal recovery. Renal recovery of kidney function was defined as free of renal replacement therapy after discontinuation of CRRT.

### 2.3. Data Collection

Demographic data, clinical characteristics, treatment details, laboratory parameters, and outcomes of interest were extracted from electronic medical chart records (MetaVision 5, iMDsoft, Tel Aviv, Israel) of the patients admitted to our ICU. All available time points (for blood gas analysis and/or routine laboratory values including electrolytes) were considered.

### 2.4. Statistical Analysis

Data were compared between the three anticoagulation groups using Pearson’s Chi-squared or Fisher’s exact test for categorical data, depending on the expected frequencies, ANOVA for normally distributed continuous data, and Kruskal–Wallis test for asymmetrical distributions. We reported the absolute and relative frequencies for categorical data, mean ± standard deviation (SD) for normally distributed data, and median [interquartile range] for asymmetrical distributions. Normality was assessed based on graphical representations. In case of statistically significant difference, post hoc pairwise comparisons were performed with Pearson’s Chi-squared or Fisher’s exact tests for categorical data, t-tests after ANOVA, and Dunn’s test after Kruskal–Wallis, with the *p*-values adjusted using the Bonferroni correction. The differences between other groups were analyzed using the same tests. The Kaplan–Meier method was used to plot the filter life according to the anticoagulation groups. The parameters measured repeatedly over time were analyzed using a mixed-effects linear model with a random intercept at the patient level. From this model, we estimated means in groups with 95% confidence intervals (CI). All-cause mortality in ICU as well as renal recovery was analyzed using binomial logistic regression according to potential predictors. We reported odds ratios (OR) with 95% CI. For the multivariable analysis, we used a stepwise procedure with a probability of entry of 0.05 and a probability of removal of 0.10 to select the variables remaining in the final model. The significance level was set at 0.05. The analyses were performed using Stata/SE 18.0 and R version 4.1.2.

## 3. Results

### 3.1. Patient’s Demographics

During the study period, 829 patients with AKI requiring CRRT were included. Of these, 552 patients had received RCA, UFH was used in 232 patients, and NA in 45 patients Reasons for not adopting citrate were due to the unavailability of citrate (6.1%), liver failure (36.5%), requirement of continuous anticoagulation for medical reason (31.8%), preference of the attending physician (2.5%), cardiogenic shock (17.7%), and bleeding and/or coagulation disorder (5.4%). Baseline characteristics on ICU admission are presented in Tables S1 and S2 (in Supplementary Materials). Overall, 567 (68.4%) patients were male; with a median age of 64 years [IQR, 55–73] and a median weight of 80 kg [IQR 70–90]. The median Sequential Organ Failure Assessment score (SOFA), a validated score for multiple organ failure in the critically ill, was 12 [IQR 9–14]. The main reason for ICU admission was medical (435 patients; 52.5%), followed by surgical (377 patients; 45.5%) with trauma patients being the least common in this population (17 patients; 2.1%). Continuous venovenous hemofiltration (CVVH) was performed in most cases (*n* = 821; 99%) while continuous venovenous hemodiafiltration (CVVHDF) was used in seven patients (0.8%) and continuous venovenous hemodialysis (CVVHD) in one patient (0.1%). Throughout the study period, a total of 1115.2 CRRT treatment days were recorded, utilizing 2926 filters.

### 3.2. Primary Outcomes

#### 3.2.1. Efficacy

**Figure 1:**
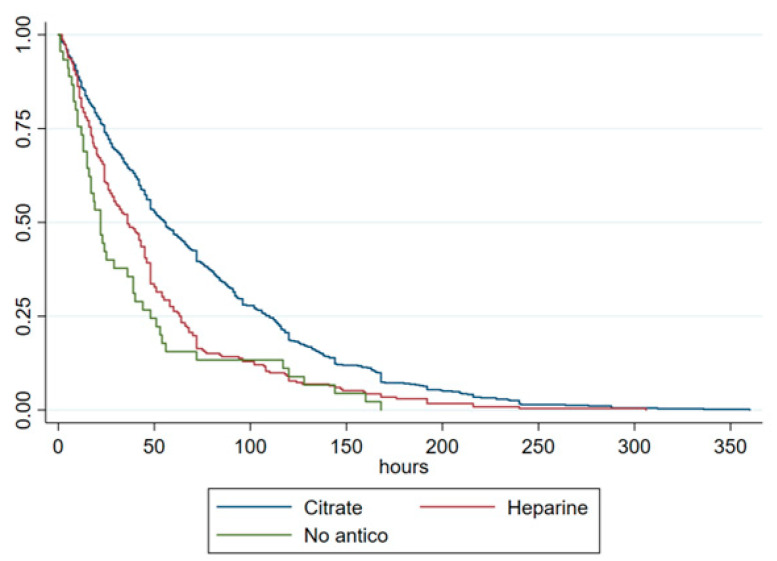
Kaplan–Meier curves of filter life for the three anticoagulation groups.

Filter lifespan was significantly longer in the RCA group with a median of 56.0 h [IQR, 24–110 h] compared to the UFH group at 36.0 h [IQR, 17–63.5 h] and the NA group at 22.0 h [IQR, 12–48 h]; *p* < 0.001. There was no significant difference in filter lifespan between UFH and NA groups (Table 1 and Figure 1). This difference could not be explained by the reason for the circuit interruption of the first filter (*p* = 0.19) or by the location of the vascular access (*p* = 0.56). Patients in the surgical group had a longer filter lifespan compared to those in the medical group (48.0 h [IQR, 24–108 h] vs. 43.0 h [IQR, 16–84 h]; P_adj_ < 0.002) (Table S3 in Supplementary Materials).

The longer filter lifespan in the RCA group compared to the UFH group could not be explained by the pre-dilution hemofiltration, as the fraction of pre-dilution was higher in the heparin group than in the RCA group. The pre-dilution was 50.0% [IQR, 50–100%] in the UFH group vs. 50.0% [IQR, 30–90%] in the RCA group; P_adj_ < 0.001. The pre-dilution was 50.0% [30.0–50.0%] in the NA group vs. 50.0% [IQR, 30–90%] in the RCA group; P_adj_ = 0.02. The difference was not significant between the NA group vs. the UFH group; P_adj_ = 0.08.

The blood flow adopted was significantly higher in the UFH group compared to the RCA group (mean 154.6 mL/min [150.3–159.0 mL/min] vs. 142.7 mL/min [140.0–145.5 mL/min]; P_adj_ < 0.001) but was not significantly different between the NA and RCA groups, nor between the NA and UFH groups.

#### 3.2.2. Safety

- Bleeding

The number of patients who required transfusion during the entire treatment period was not significantly different between the groups. However, the number of packed cells transfused was significantly lower in the RCA group compared to the UFH group (median 3 units [IQR, 2–7 units] vs. median 5 units [IQR, 2–12 units]; P_adj_ < 0.001) and fewer in the NA group compared to the UFH group (median 3 units [IQR, 1–5 units] vs. 5 units [IQR, 2–12 units]; P_adj_ = 0.03). The incidence of overt/active bleeding was similar across groups (31.2% in the RCA group vs. 40.3% in the UFH group and 41.2% in the NA group; *p* = 0.08). The number of packed cells transfused in patients with overt bleeding was also not significantly different (median 8 units [IQR, 4–16] in the RCA group vs. median 9 units [IQR, 5–34] in the UFH group and 5.5 units [IQR, 2–11] in the NA group; *p* = 0.06) (Table 2).

Extracorporeal life support requires anticoagulation with unfractionated heparin. The addition of heparin in those patients on ECLS presenting with overt bleeding increased the need for transfusions (69 patients in the ECLS group received a median of 28 units [IQR, 12–42 units] vs. 127 patients in the no ECLS group, median 6 units [IQR, 3–9 units]; *p* < 0.001). Patients in the RCA group who received heparin for ECLS had higher transfusion requirements compared to those who received citrate alone (median 22 units [IQR, 10–37 units] vs. 6 units [IQR, 4–10]; *p* < 0.001) (Table S4 in Supplementary Materials).

- Acid–base disorders

The prevalence of metabolic alkalosis was high, affecting up to 22.5% of patients after 72 h of treatment, and increasing to 32.5% by the end of CRRT in the RCA group, compared to 16.2% in the UFH group 13.5% in the NA group (*p* < 0.001). Metabolic acidosis was observed in 3.0% of patients after 72 h and increased to 16.8% by the end of therapy in the citrate group. For patients in the UFH group, the prevalence of metabolic acidosis was 6.3% after 72 h and rose to 29.1% by the end of therapy. Patients in the UFH group were statistically more acidotic compared to those in the RCA group by the end of therapy (P_adj_ < 0.001). Additionally, patients in the NA group remained more acidotic at the end of therapy compared to the RCA group (P_adj_ = 0.006), with a prevalence of 34.6% compared to 16.8% (Table 2). These acid–base derangements were also reflected by the apparent strong ion difference (SIDa). SIDa at the beginning of treatment was equal between groups (*p* = 0.162). However, after 72 h of treatment, SIDa was significantly higher in the RCA group compared to the UFH group (39.56 ± 4.58 vs. 35.95 ± 3.9; P_adj_ = 0.0001) and compared to the NA group (SIDa of 39.56 ± 4.58 vs. 36.11 ± 2.15; P_adj_ = 0.025), but not significantly different between the UFH group and NA group (P_adj_ = 1.00) (Table S5 in Supplementary Materials).

- Electrolyte disorders

The prevalence of electrolyte disorders, including hypocalcemia (RCA 2.5%, UFH 0%, NA 0%), hypokalemia (RCA 14.6%, UFH 15.6%, NA 3.5%), hypomagnesemia (RCA 38.5%, UFH 25.0%, NA 33.3%), hypophosphatemia (RCA 30.5%, and UFH 34.5%, NA 25.0%), was similar across all groups (Table 2).

However, the incidence of hyponatremia was higher in the citrate group (18.8% in the RCA group vs. 6.9% in the UFH group and 0% in the NA group; *p* < 0.001). No cases of hypernatremia were encountered during treatment.

Citrate accumulation (CA), defined as a total/ionized calcium ratio ≥ 2.5, was observed in 7.6% of the citrate group, compared to 3% in the heparin group, and 4.4% in the no anticoagulation group (*p* = 0.045). In patients who presented with citrate accumulation, a mortality rate of 82.3% was detected (Table S6 in Supplementary Materials).

- Secondary outcomes

Regarding renal outcome, there was a significant difference in dialysis independence among patients, with 186 (33.6%) patients in the RCA group, 52 (22.5%) in the UFH group, and 8 (17.8%) in the NA group. This was statistically significant for citrate versus heparin (P_adj_ = 0.006). However, there was no significant difference between the heparin versus no anticoagulation groups, or between citrate and no anticoagulation groups (Table 1).

All-cause mortality in the ICU was significantly lower in the RCA group (52.7%) compared to the UFH group (63.4%); P_adj_ = 0.02 and the NA group (77.8%); P_adj_ = 0.003, but there was no significant difference between the UFH and the NA groups. A higher SOFA score in the NA group could explain the higher mortality, but this was not the case for the heparin versus the RCA group (Table 1).

A longer filter life, anticoagulation with citrate, and patients admitted for trauma all were related to an increased chance of renal recovery (Table 3), whereas transfusion of RBC was not. Regarding the mortality in ICU (Table 4), several variables seemed to have an effect when analyzed separately. We built a multivariable logistic regression model keeping only variables significantly associated with mortality and found that a longer filter life, anticoagulation with citrate, and the presence of diabetes mellitus were associated with a lower risk of death, whereas no anticoagulation, a higher SAPS score, and APACHE IV were associated with a higher risk of mortality.

A total of 2926 filters were used during the study period, with a total CRRT duration of 26,765 h. The total duration of CRRT was significantly longer for the RCA group compared to the NA group (median 168 h [IQR, 72–336 h] vs. median 72 h [IQR, 32–144 h]; P_adj_ < 0.001); and for the UFH group compared to the NA group (median 144 h [IQR, 42.5–336 h] vs. median 72 h [IQR, 32–144 h]; P_adj_ = 0.004).

Patients who developed severe hypophosphatemia after 72 h of treatment required significantly more time on the ventilator (median 34 days [IQR, 22–50 days] vs. median 27.5 days [IQR, 18–42 days]; *p* = 0.02). (Table S7 in Supplementary Materials) Additionally, 54 patients showed signs of citrate overdose (total/ionized calcium ratio ≥ 2.5 and metabolic alkalosis).

## 4. Discussion

In this to our knowledge largest conducted retrospective analysis, we shed some light on the efficacy and safety of various anticoagulation strategies during continuous renal replacement therapy. Our analysis revealed several noteworthy observations. First, we found that the time to circuit failure is significantly longer while using citrate, with fewer bleeding complications. Regional citrate anticoagulation was associated with metabolic alkalosis and hyponatremia. The prevalence of hypocalcemia was infrequent, and its occurrence rate was like the other anticoagulation methods. However, electrolytes such as phosphate and magnesium, which are not standard components of the CRRT fluids, provoke a high incidence of hypophosphatemia and hypomagnesemia, which can have significant negative implications on morbidity and mortality. Mortality was significantly lower in the RCA group.

### 4.1. Efficacy

- Prolonged filter life with citrate

Like previous studies [6], our data demonstrate that citrate is superior to heparin in terms of filter patency. The extended filter life and reduced need for circuit changes during RCA highlight its efficacy in maintaining filter patency and preventing clot formation within the CRRT system. Reducing circuit interruptions reduces blood loss and associated costs, reduces downtime, and increases treatment time. This finding cannot be attributed to the reasons for circuit disconnections or the use of pre-dilution, which may reduce membrane clotting compared to post-dilution in pure convective modes [11] as the UFH group and the NA group had a significantly higher pre-dilution fraction than the RCA group.

Other non-anticoagulant measures, such as the site of vascular access in relation to filter life, are still under debate. Brain et al. [12] favored femoral access over internal jugular and subclavian access as last. A more recent Cochrane systematic review by Tsjujimoto et al. [13] suggested that the right jugular access does not have a clear advantage over femoral access in terms of filter life. Our results align with this finding, indicating that the choice of puncture site did not impact filter survival.

For patients with a (relative) contraindication to citrate (i.e., liver failure) or heparin (i.e., increased risk of bleeding), CRRT was performed without anticoagulation. Consistent with the findings of a recently published systematic review and meta-analysis, our study did not reveal any significant difference in filter lifespan between the anticoagulation-free and systemic heparin group [14]. This challenges the conventional practice of routinely administering anticoagulation during CRRT in certain patient populations, particularly those with a high risk of bleeding. Anticoagulation with heparin may not provide additional benefits for CRRT circuit longevity.

Furthermore, we observed a significant difference in filter life based on the reason for admission. Patients admitted for medical reasons experienced a shorter filter life than those admitted for surgical reasons, though this association was not observed in trauma patients. The reasons underlying these discrepancies warrant further investigation but may be related to differences in baseline coagulation status, severity of illness, or the underlying pathophysiological mechanism in medical and surgical patient populations.

### 4.2. Safety

- Less bleeding complications with citrate

Consistent with previous research, our findings reveal a lower incidence of bleeding complications in patients receiving citrate anticoagulation compared to those receiving heparin anticoagulation [5,15,16]. Citrate anticoagulation exerts its anticoagulant effect by chelating calcium ions, thereby inhibiting the coagulation cascade without directly affecting platelet function [17] This mechanism may contribute to the reduced risk of bleeding, making citrate an attractive option for patients at high risk of hemorrhagic complications.

The number of packed cells transfused was significantly lower in patients receiving citrate compared to those in the heparin group. However, this difference was not reflected in the overall number of patients who required transfusion, or in those patients who experienced bleeding complications necessitating an additional intervention. A possible explanation for this discrepancy is that almost all patients who are admitted to the ICU receive prophylactic anticoagulation or even therapeutic anticoagulation with systemic unfractionated heparin for extracorporeal life support (ECLS). In fact, a subgroup analysis of the patients in the RCA group who suffered overt bleeding showed that 30% were on ECLS with UFH and received considerably more transfusion than patients without ECLS.

- Acid–base disturbances

Citrate chelates ionized calcium, a crucial cofactor in the coagulation cascade. When its ionic form drops below 0.35 mmol/L, anticoagulation is achieved. Citrate not removed by the hemofilter returns to the patient and is metabolized in the liver, muscle, and kidney, entering the Krebs cycle and generating three molecules of sodium bicarbonate per citrate molecule. If this process occurs, RCA leads to plasma alkalinization [6,18]. This can also be explained by the Stewart–Figge method, which postulates that acid–base balance and pH depend on the difference between the concentrations of strong cations and strong anions (i.e., the strong ion difference; SID), the PaCO2, and the total concentration of weak acids. Stewart introduced the term apparent SID (SIDa), calculated as ([Na^+^] + [K^+^] + [Mg^2+^] + [Ca^2+^]) − ([Cl^−^] + [lactate^−^]) [19]. The normal range for SIDa is approximately 40–44 mmol/L [19]. The increased SIDa (54 mmol/L) of Prismocitrate 18 leads to alkalinization [20].

SIDa at the beginning of treatment was equal between groups. However, after 72 h of treatment, SIDa was significantly higher in the RCA group compared to the UFH group and NA group, but not significantly different between the UFH group and NA group.

Excessive citrate administration or decreased extracorporeal clearance, such as from clogging, can result in net citrate overload, causing metabolic alkalosis without hypocalcemia [7]. While the prevalence of metabolic alkalosis after 72 h of treatment did not differ between groups, this effect became more pronounced with longer treatment duration, showing a significantly increased prevalence of alkalosis in the citrate group. The reported incidence of metabolic alkalosis associated with RCA is higher than previously noted in prior studies (2.4–8.3%) [21,22,23]. A possible explanation for this finding is that during the study period, we did not use low bicarbonate-containing replacement solutions and even if we use postfilter monitoring to adapt citrate administration, our protocol does not include an algorithm for therapy adaptation to normalize these values.

The body’s capacity to metabolize citrate is saturable and may be reduced in case of liver failure, hypoperfusion states with decreased oxygen delivery for proper functioning of the Krebs cycle (typically occurring in multiple organ failure), and intoxications causing mitochondrial blockade [24]. Citrate accumulation, potentially lethal, can be identified by a total calcium/ionic calcium ratio ≥ 2.5, a progressive increase in intravenous calcium infusion requirements, and the presence of progressive metabolic acidosis with a high anion gap [18,25,26]. The prevalence of citrate accumulation was low (*n* = 51 patients), but significantly higher in the citrate group. Link et al. showed that patients with a Cat/Cai ratio ≥ 2.4 had a 33-fold increased risk of death compared to those with lower values [27].

In our study, 79.2% of patients with CA had liver failure, 92.2% had a high lactate level as a sign of shock and low tissue perfusion and 82.3% of those patients who experienced citrate accumulation died.

- Electrolyte disturbances

RRT treatment can result in unintended electrolyte complications, particularly in patients undergoing prolonged or high-dose RRT [28,29]. The prevalence of electrolyte derangements is high, with a reported incidence of up to 67% [30,31,32]. Hypophosphatemia and hypomagnesemia are the most common issues, as commercially available replacement fluids do not typically contain phosphate or magnesium [31]. Hypophosphatemia can lead to respiratory muscle dysfunction, and failure to wean from mechanical ventilation. The overall prevalence of hypomagnesemia (35.2%) and hypophosphatemia (31%) in our study was high. While there was no significant difference between groups, there was a clear association between hypophosphatemia and a longer duration of invasive mechanical ventilation.

The incidence of hyponatremia was higher in the citrate group (18.8% in the RCA group vs. 6.9% in the UFH group and 0% in the NA group; *p* < 0.001). A possible explanation for the hyponatremia observed in the citrate group may lie in the administration of the high-volume hypotonic citrate solution (Prismocitrate 18; osmolarity: 244 mosm/L). No cases of hypernatremia were encountered during treatment.

During RCA-CRRT, approximately, 30–70% of the calcium–citrate complex is removed by convection or diffusion [33]. The incidence of hypocalcemia in our population was low and, unlike previous studies, was not higher in the citrate group, confirming that with the help of a strict protocol, complications of hypocalcemia can be avoided.

- Secondary outcomes

An unexpected finding was that a longer filter life increased the chance of renal recovery and was higher in patients in the citrate anticoagulation group. Moreover, the chance of renal recovery was higher if the admission reason was trauma compared to medical. The number of packed cells did not seem to have a significant effect on renal recovery, mortality, or filter life.

All-cause mortality was significantly lower in the citrate group compared to the heparin group and anticoagulation-free group. Recent results from meta-analysis failed to demonstrate the impact of different anticoagulation strategies on mortality [5].

The survival benefit appeared to be linked to citrate anticoagulation, longer filter lifespan, lower severity of illness (indicated by lower SAPS and APACHE IV scores), and surprisingly, the presence of diabetes. There are several hypothetical explanations for this. It is possible that a longer filter life span in the citrate group, resulting in higher effectiveness of the therapy, resulted in this outcome effect. Trauma patients show higher renal recovery rates, likely because they are generally younger and have fewer comorbidities compared to medical patients. The presence of diabetes might be due to better-managed care, surprisingly contributing to improved survival in the context of this study. The insignificance of packed cells transfusion on renal recovery, filter life, and mortality might be related to the fact that patients who receive packed cells might have varying underlying conditions that mask the potential benefits or harms of transfusion on renal recovery, mortality, or filter life. Further research would be necessary to confirm these hypotheses and better understand the causal relationships.

Although heparin-induced thrombocytopenia (HIT) can be potentially serious and difficult to diagnose in critically ill patients [34], the prevalence was rare and did not differ between groups.

Our study has several limitations. The retrospective, single-center design may introduce bias and limit generalizability. Complications were not predefined, and documentation is subject to human error. However, all data are automatically entered in our electronic medical chart system, minimizing the risk of data loss. Our protocol mandates routine nursing surveillance for patients undergoing CRRT, including multiple daily arterial blood gas analyses and electrolyte level measurements as part of regular follow-up. Additionally, our protocol requires regular monitoring of systemic and total ionized calcium. The method of anticoagulation is not protocolized and is based on the physician’s preferences and experience, introducing selection bias. Complications, such as transfusion requirements, may be influenced by the non-standardized transfusion trigger and even by blood loss from routine ICU sampling. Despite these limitations, our analysis included a substantial number of patients over a long time period, reflecting daily practice rather than study purpose.

## 5. Conclusions

Regional citrate anticoagulation resulted in a longer filter life compared to heparin and no anticoagulation. The benefit of a longer filter lifespan may lead to improved outcomes through higher therapy effectiveness and reduced transfusion events. The prevalence of electrolyte derangements, such as hypomagnesemia and hypophosphatemia, is high and a consequence of CRRT rather than the anticoagulation method used. Metabolic alkalosis and hyponatremia were linked to citrate use. Monitoring remains crucial to limit potential complications attributable to its use.

## Figures and Tables

**Table 1:** Filter life and CRRT parameters.

	All (*n* = 829) *n* (%) Median [IQR]	RCA (*n* = 552) *n* (%) Median [IQR]	UFH (*n* = 232) *n* (%) Median [IQR]	NA (*n* = 45) *n* (%) Median [IQR]	*p*-Value	RCA vs. UFH *p*-Value_adj_	RCA vs. NA *p*-Value_adj_	UFH vs. NA *p*-Value_adj_
Survival time first filter, h	46 [20–92]	56 [24–110]	36 [17–63.5]	22 [12–48]	<0.001	<0.001	<0.001	0.13
Place of puncture					0.35			
Femoral	408 (49.2)	259 (46.9)	121 (52.2)	28 (62.2)				
Right jugular	198 (23.9)	134 (24.3)	56 (24.1)	8 (17.8)				
Left jugular	198 (23.9)	140 (25.4)	49 (21.1)	9 (20.0)				
Subclavian	25 (3.0)	19 (3.4)	6 (2.6)	0				
Modality of CRRT					0.046	0.03	1.00	1.00
CVVH	821 (99.0)	550 (99.6)	226 (97.4)	45 (100.0)				
CVVHD	1 (0.1)	0	1 (0.43)	0				
CVVHDF	7 (0.8)	2 (0.4)	5 (2.2)	0				
Number of circuits	2 [1–5]	2 [1–4]	3 [1–5]	2 [1–4]	0.009	0.004	1.00	0.13
Reason for circuit interruption first filter:					0.19			
Circuit clotting	509 (61.4)	324 (58.7)	152 (65.8)	33 (73.3)				
death	109 (13.1)	67 (12.1)	32 (13.9)	10 (22.2)				
logistic reasons	97 (11.7)	68 (12.3)	27 (11.7)	2 (4.4)				
Technical problems	12 (1.5)	10 (1.8)	2 (0.9)	0				
human error	10 (1.2)	6 (1.1)	4 (1.7)	0				
catheter dysfunction	31 (3.7)	25 (4.5)	6 (2.6)	0				
unclear	13 (1.6)	11 (2.0)	2 (0.9)	0				
Recovery of kidney function/diuresis	26 (3.1)	23 (4.0)	3 (1.3)	0				
stop of therapy for any reason	13 (1.6)	10 (1.8)	3 (1.3)	0				
change in anticoagulation	6 (0.7)	6 (1.1)	0	0				
due to no anticoagulation	1 (0.1)	1 (0.2)	0	0				
change in CRRT mode	2 (0.2)	2 (0.4)	0	0				
Total duration of CRRT, h	144 [57–336]	168 [72–336]	144 [42.5–336]	72 [32–144]	<0.001	0.16	<0.001	0.004
Total duration of CRRT, d	6 [2.4–14]	7 [3–14]	6 [1.9–14]	3 [1.3–6]				
Recovery of kidney function	246 (29.7)	186 (33.6)	52 (22.5)	8 (17.8)	0.002	0.006	0.09	1.00

RCA: regional citrate anticoagulation; UFH: unfractionated heparin; NA: no anticoagulation; CRRT: continuous renal replacement therapy; CVVH: continuous venovenous hemofiltration; CVVHD: continuous venovenous hemodialysis; CVVHDF: continuous venovenous hemodiafiltration.

**Table 2:** Adverse events.

Variables	RCA		UFH		NA		*p*-Value	RCA vs. UH P_adj_	RCA vs. NA P_adj_	UH vs. NA P_adj_
	*n*	*n* (%) Median [IQR]	*n*	*n* (%) Median [IQR]	*n*	*n* (%) Median [IQR]				
Bleeding complications								<0.001	0.74	0.03
Patients transfused	553	367 (66.4)	231	172 (74.5)	45	34 (75.6)	0.052			
n of PRBC	367	3 [2–7]	172	5 [2–12]	34	3 [1–5]	0.001			
Patients with overt bleeding	362	113 (31.2)	171	69 (40.3)	34	14 (41.2)	0.08			
n of PRBC if overt bleeding	113	8 [4–16]	69	9 [5–34]	14	5.5 [2–11]	0.06			
HIT	552	4 (0.7)	232	4 (1.7)	45	0	0.42			
After 72 h								0.003	0.03	0.65
Acidosis (pH < 7.3)	404	12 (3.0)	144	9 (6.3)	29	1 (3.5)	0.21			
Alkalosis (pH > 7.5)	404	91 (22.5)	144	24 (16.7)	29	4 (13.8)	0.21			
Hyponatremia	404	76 (18.8)	144	10 (6.9)	29	0	< 0.001			
Hypocalcemia	404	10 (2.5)	144	0	29	0	0.17			
Hypokalemia	404	59 (14.6)	144	21 (15.6)	29	1 (3.5)	0.24			
Hypomagnesemia	169	65 (38.5)	52	13 (25.0)	12	4 (33.3)	0.20			
Hypophosphatemia	177	54 (30.5)	58	20 (34.5)	12	3 (25.0)	0.76			
At the end of therapy	529		210		52					
Acidosis (pH < 7.3)		89 (16.8)		61 (29.1)		18 (34.6)	<0.001	<0.001	0.006	1.00
Alkalosis (pH > 7.5)		172 (32.5)		34 (16.2)		7 (13.5)	<0.001	<0.001	0.015	1.00
Citrate accumulation	552	42 (7.6)	232	7 (3.0)	45	2 (4.4)	0.045	0.045	1.00	1.00

Ionized calcium level < 0.85 mmol/L, sodium < 130 mmol/L, Potassium < 3.5 mmol/L, Magnesium < 0.66 mmol/L, phosphate < 0.78 mmol/L, PRBC: packed red blood cells, HIT: heparin-induced thrombocytopenia, RCA: regional citrate anticoagulation, NA: no anticoagulation.

**Table 3:** Variables associated with renal recovery.

Variables	Univariable			Multivariable (*n* = 826)	
	*n*	Renal Recovery OR [95% CI]	*p*-Value	Renal Recovery OR_adj_ [95% CI]	*p*-Value
Filter life (hours)	826	1.003 [1.001; 1.006]	0.008	1.003 [1.000; 1.005]	0.047
Anticoagulation	829		0.002		0.04
RCA		1		1	
Heparin		0.57 [0.40; 0.82]		0.64 [0.45; 0.92]	
NA		0.43 [0.19; 0.93]		0.60 [0.28; 1.29]	
Admission reason	826		0.03		0.04
Medical		1		1	
Surgical		1.32 [0.97; 1.79]		1.28 [0.94; 1.74]	
Trauma		3.10 [1.17; 8.23]		3.07 [1.15; 8.21]	

**Table 4:** Variables associated with mortality in ICU.

Variables	Univariable			Multivariable (*n* = 784)	
	*n*	Death (ICU) OR [95% CI]	*p*-Value	Death (ICU) OR_adj_ [95% CI]	*p*-Value
Filter life (hours)	830	0.995 [0.992; 0.997]	<0.001	0.996 [0.994; 0.999]	0.004
Anticoagulation	830		<0.001		0.009
RCA		1		1	
Heparin		1.55 [1.13; 2.12]		1.46 [1.03; 2.07]	
NA		3.13 [1.52; 6.44]		2.80 [1.24; 6.36]	
SAPS score	830	1.03 [1.02; 1.04]	<0.001	1.02 [1.01; 1.03]	0.004
APACHE IV score	784	1.02 [1.01; 1.02]	<0.001	1.01 [1.00; 1.02]	0.007
Diabetes	830	0.72 [0.48; 1.09]	0.12	0.62 [0.40; 0.97]	0.04

## Data Availability

All data generated or analyzed during this study are included in this article and its Supplementary Material files. Further inquiries can be directed to the corresponding author.

## Supplementary Materials

The following supporting information can be downloaded at: https://www.mdpi.com/article/10.3390/life14101304/s1, Table S1: Patient’s demographics and ICU admission characteristics; Table S2: Baseline values before start of CRRT; Table S3: Factors affecting filter lifespan; Table S4: Transfusion requirements; Table S5: Evolution of lab values after 72 h of treatment; Table S6: Impact of citrate accumulation; Table S7: Association between hypophosphatemia after 72 h of treatment and length of ventilation.
