# Supplementary material for: Optimizing Continuous Renal Replacement Therapy with Regional Citrate Anticoagulation: Insights from the ORCA Trial—A Retrospective Study on 10 Years of Practice
Source: Life (Basel). 2024 Oct 14;14(10):1304. doi: 10.3390/life14101304 (PMC11509773; doi:10.3390/life14101304)
Supplement: Supplementary file 1 [file life-14-01304-s001.zip › life-3205063-supplementary.pdf]

Table S1: Patient's demographics and ICU admission characteristics

Variables	All (n = 829)<br>n (%)<br>Median [IQR]<br>Mean ± SD	RCA (n = 552)<br>n (%)<br>Median [IQR]<br>Mean ± SD	UFH (n = 232)<br>n (%)<br>Median [IQR]<br>Mean ± SD	NA (n = 45)<br>n (%)<br>Median [IQR]<br>Mean ± SD	p-value	RCA vs. UFH<br>$p\text{-value}_{adj}$	RCA vs. NA<br>$p\text{-value}_{adj}$	UFH vs. NA<br>$p\text{-value}_{adj}$
Male	567 (68.4)	390 (70.7)	154 (66.4)	23 (51.1)	0.02	0.71	0.02	0.15
Age (years)	64 [55-73]	66 [58-74]	61 [51-71]	59 [48-67]	< 0.001	< 0.001	< 0.001	0.38
Weight (kg)	80 [70-90]	80 [70-90]	80 [70-92]	72 [62-90]	0.053	1.00	0.02	0.052
BMI (kg/m²)	26.3 [23.9-30.3]	26.3 [23.9-30.5]	26.3 [23.8-30.1]	25.4 [23.0-29.1]	0.35			
SOFA score	12 [9-14]	12 [9-14]	12 [9-15]	14 [11-17]	0.003	0.06	< 0.001	0.01
SAPS 3	64.4 ± 15.0	63.8 ± 14.7	64.5 ± 15.4	71.0 ± 15.1	0.008	1.00	0.006	0.02
APACHE II	24.7 ± 7.4	24.5 ± 7.3	24.5 ± 7.6	27.4 ± 7.1	0.04	1.00	0.03	0.05
APACHE IV	51 [26 ; 71]	50 [27 ; 69]	46 [22 ; 73]	69 [45 ; 87]	0.005	0.23	0.001	< 0.001
Reason of admission					0.08			
Medical	435 (52.5)	288 (52.2)	115 (49.6)	32 (71.1)				
Surgical	377 (45.5)	250 (45.3)	114 (49.1)	13 (28.9)				
Trauma	17 (2.1)	14 (2.5)	3 (1.3)	0				
Co-existing conditions:								
Diabetes	104 (12.5)	72 (13.0)	27 (11.6)	5 (11.1)	0.83			
Immunocompromised	85 (10.3)	66 (12.0)	13 (5.6)	6 (13.3)	0.02	0.02	1.00	0.29
Chemotherapy	54 (6.5)	39 (7.1)	11 (4.7)	4 (8.9)	0.39			
Radiotherapy	54 (6.5)	39 (7.1)	11 (4.7)	4 (8.9)	0.39			
Chronic heart failure	240 (28.9)	174 (31.5)	58 (25.0)	8 (17.8)	0.04	0.20	0.16	0.89
Liver cirrhosis	92 (11.1)	39 (7.1)	36 (15.5)	17 (37.8)	< 0.001	< 0.001	< 0.001	0.003
COPD	50 (6.0)	34 (6.2)	13 (5.6)	3 (6.7)	0.94			
Chronic renal failure	213 (25.7)	159 (28.8)	45 (19.4)	9 (20.0)	0.01	0.02	0.62	1.00
Arterial hypertension	405 (48.9)	297 (53.8)	95 (40.9)	13 (28.9)	< 0.001	0.003	0.003	0.39
LOS (days)	18.4 [8.8-18.4]	20.9 [10.6-37.9]	16.0 [7.5-30.8]	10.4 [2.8-23.9]	< 0.001	0.02	< 0.001	0.04
Length of ventilation (days)	12 [5-26]	13 [5-27]	11 [4-23.5]	7 [3 ; 14]	0.02	0.49	0.008	0.04
ICU outcome: death	473 (57.1)	291 (52.7)	147 (63.4)	35 (77.8)	< 0.001	0.02	0.003	0.19
Medical	282 (59.6)	176 (60.5)	79 (53.7)	27 (77.1)				
Surgical	183 (38.7)	110 (37.8)	65 (44.2)	8 (22.9)				
Trauma	8 (1.7)	5 (1.7)	3 (2.1)	-				
Hospital outcome: death	522/824 (63.3)	327/547 (59.8)	158 (68.1)	37 (82.2)	0.002	0.08	0.009	0.17

RCA: regional citrate anticoagulation; UFH: unfractionated heparin; NA: no anticoagulation; ICU: intensive care unit; IQR: interquartile range; BMI: body mass index; SOFA: sequential organ failure; SAPS: Simplified Acute Physiology Score; APACHE: Acute Physiology And Chronic Health Evaluation; COPD: chronic obstructive pulmonary disease: LOS, length of stay

Table S2: Baseline values before start of CRRT

Variables	RCA		UFH		NA		p-value	RCA vs. UH	RCA vs. NA	UH vs. NA
	n	mean ± SD median [IQR]	n	mean ± SD median [IQR]	n	mean ± SD median [IQR]		p_{adj}	p_{adj}	p_{adj}
**Blood gas**	549		225		55					
pH		7.34 ± 0.11		7.34 ± 0.11		7.29 ± 0.15	0.003	1.000	0.002	0.009
Bicarbonate		21.59 ± 4.92		20.81 ± 5.31		19.01 ± 5.46	0.001	0.152	0.001	0.056
$PaCO_2$		40.5 ± 10.0		39.2 ± 10.0		39.9 ± 11.1	0.270			
Base excess		-3.8 ± 5.6		-4.6 ± 6.1		-7.0 ± 7.0	0.000	0.299	0.000	0.017
Lactate		2.1 [1.3 - 4.3]		2.8 [1.7 - 8.8]		5.7 [2.1 - 13]	0.000	0.000	0.000	0.002
**SIDa**	505	36.65±5.73	191	35.79±6.13	42	35.63±6.94	0.162			
**Electrolytes**										
Ionized Calcium	549	0.99 ± 0.14	225	1.04 ± 0.11	55	1.08 ± 0.15	0.000	0.000	0.000	0.161
Total Calcium	523	2.13 ± 0.25	209	2.02 ± 0.20	43	2.13 ± 0.22	0.000	0.000	1.000	0.013
Magnesium	505	0.84 ± 0.25	192	1.07 ± 0.25	43	1.04 ± 0.23	0.000	0.000	0.000	1.000
Phosphate	523	1.51 ± 0.66	209	1.52 ± 0.72	43	1.52 ± 0.93	0.963			
Sodium	549	135.3 ± 6.2	225	136.9 ± 6.8	55	138.3 ± 6.4	0.000	0.003	0.002	0.435
Chloride	549	102.1 ± 6.1	225	103.7 ± 5.9	55	103.1 ± 5.1	0.003	0.003	0.695	1.000
Potassium	549	4.38 ± 0.88	225	4.66 ± 0.92	55	4.58 ± 0.86	0.000	0.000	0.332	1.000
**Kidney function**	534		220		50					
Creatinin		2.66 [1.8 -3.91]		2.62 [1.82 - 4.03]		2.07 [1.45 - 3.06]	0.134			
Ureum		110 [74 - 163]		108 [72 - 155]		91.5 [62 - 146]	0.100			
**CRRT values**										
bloodflow	549	141.1 ± 21.1	223	150.0 ± 67.6	53	147.2 ± 20.2	0.015	0.014	0.864	1.000
ultrafiltration rate	549	1616 ± 781	222	1867 ± 533	54	1852 ± 594	0.000	0.000	0.061	1.000
PRE replacement	544	50 [20 - 50]	221	50 [50 - 50]	53	50 [50 - 50]	0.000	0.000	0.000	1.000
**Coagulation parameter**										
Hemoglobin	537	8.6 ± 1.5	219	8.9 ± 1.5	51	8.6 ± 1.1	0.045	0.047	1.000	0.455
Platelets	537	114 [69 - 191]	218	107 [66 - 177]	51	81 [51 - 163]	0.043	0.217	0.133	1.000
aPTT	534	40.3 [32 - 52.9]	217	60.7 [44.1 - 84.3]	52	57.5 [41.1 - 84.3]	0.000	0.000	0.001	1.000
Calcium postfilter	528	0.34 [0.29 - 0.41]								

RCA: regional citrate anticoagulation; UFH: unfractionated heparin; NA: no anticoagulation; SIDa: strong ion difference apparent

Table S3: Factors affecting filter lifespan

	n	Duration of the first filter (hours) Median [IQR]	p-value	M vs. S p-value$_{adj}$	M vs. T p-value$_{adj}$	S. vs. T p-value$_{adj}$
Place of puncture			0.56			
Femoral	408	48 [18-91.5]				
Jugular right	198	43.5 [22-81]				
Jugular left	198	48 [20-106]				
Subclavian	25	48 [24-65]				
ICU admission			0.002	0.002	0.62	0.12
Medical	435	43 [16-84]				
Surgical	377	48 [24-108]				
Trauma	17	32 [18-55]				

IQR: interquartile range; M: medical; S: surgical; T: trauma

Table S4 : Transfusion requirements

	n	N of PRBC Median [IQR]	p-value
Patients in RCA-group with any overt bleeding			< 0.001
ECLS+ (+UFH)	34	22 [10-37]	
ECLS-	79	6 [4-10]	
Patients with any overt bleeding +ECLS (UFH)			< 0.001
No	127	6 [3-9]	
Yes	69	28 [12-42]	
Patients with any overt bleeding receiving UFH			< 0.001
No	87	6 [3-10]	
Yes	109	12 [6-34]	

PRBC: packed red blood cells, ECLS: extracorporeal life support, RCA: regional citrate anticoagulation, UFH: unfractionated heparin

Table S5: Evolution of lab values after 72 hours of treatment

	RCA		UFH		NA		p-value	RCA vs. UH p-value adj	RCA vs. NA p-value adj	UH vs. NA p-value adj
**Variables**	n	mean±SD	n	mean±SD	n	mean±SD				
**Blood gas**	404		144		29					
pH		7.45±0.07		7.41±0.08		7.42±0.06	0.000	0.000	0.274	1.000
Bicarbonate		27.64±3.76		24.63±3.06		24.73±3.34	0.000	0.000	0.000	1.000
$PaCO_2$		41.03±7.38		39.76±7.39		38.78±5.67	0.078			
Base excess		3.36±3.96		0.09±3.47		0.38±3.45	0.000	0.000	0.000	1.000
Lactate		1.85±2.21		2.35±2.52		2.36±2.11	0.054			
**SIDa**	167	39.56±4.58	52	35.97±3.9	12	36.11±2.15	0.000	0.000	0.025	1.000
**Electrolytes**										
Ionized Calcium	404	1.11±0.15	144	1.18±0.11	29	1.20±0.11	0.000	0.000	0.003	1.000
Total Calcium	177	2.29±0.22	58	2.21±0.24	12	2.17±0.31	0.031	0.087	0.247	1.000
Magnesium	169	0.73±0.21	52	0.79±0.2	12	0.80±0.19	0.178			
Phosphate	177	0.99±0.37	58	1.08±0.5	12	1.18±0.4	0.134			
Sodium	404	132.68±2.88	144	134.39±2.81	29	134.44±2.27	0.000	0.000	0.004	1.000
Chloride	404	98.16±3.96	144	103.18±3.30	29	103.17±3.4	0.000	0.000	0.000	1.000
Potassium	404	3.89±0.45	144	4.15±0.61	29	4.31±0.5	0.000	0.000	0.000	0.313
**Kidney function**	250		98		17					
Creatinine		1.22±0.56		1.52±0.74		1.41±0.89	0.000	0.000	0.717	1.000
Ureum		61.15±28.78		75.16±32.15		80.88±39.62	0.000	0.000	0.029	1.000
**CRRT values**	402		132		27					
Bloodflow		143.73±21.63		149.85±23.38		150.37±22.95	0.012	0.018	0.395	1.000
ultrafiltration rate		1482.06±879.55		1872.50±509.61		1852.37±480.17	0.000	0.000	0.057	1.000
PRE replacement		30.41±22.16		50.91±14.42		43.89±20.95	0.000	0.000	0.003	0.319
**Coagulation parameter**										
Hb										
Platelets	276	8.57±1.09	100	8.67±1.23	20	8.72±00.83	0.678			
aPTT	276	167.04±145.58	100	115.06±106.65	20	82.60±53.67	0.000	0.003	0.020	0.965
postfilter Calcium	270	40.14±23.34	110	59.48±42.82	21	58.96±29.07	0.000	0.000	0.019	1.000
	311	0.36±0.17	47	0.60±0.41	7	0.52	0.000	0.000	0.182	1.000

RCA: regional citrate anticoagulation, UFH: unfractionated heparin, NA: no anticoagulation, Hb: hemoglobin, SIDa: strong ion difference apparent

Table S6: Impact of citrate accumulation

**Variables**	**Citrate accumulation (n=51) n (%)**
Outcome (death)	42 (82.3)
Lactate	47 (92.2)
Liver dysfunction	42 (79.2)

Table S7: Association between hypophosphatemia after 72 hours of treatment and length of ventilation

	n	Length of ventilation (days) Median [IQR]	p-value
Hypophosphatemia			0.02
No	170	27.5 [18 ; 42]	
Yes	77	34 [22 ; 50]	
